# Urinary monocyte chemoattractant protein-1 levels and interstitial changes in the renal cortex and their relationship with loss of renal function in renal transplant patients with delayed graft function

**DOI:** 10.1186/s40697-015-0038-9

**Published:** 2015-01-30

**Authors:** Miguel Moyses Neto, Elen A Romão, Gyl EB Silva, Marcio Dantas, Maria EP Nardim, Sylvio Tucci, Heloísa DC Francescato, Terezila M Coimbra

**Affiliations:** Department of Internal Medicine, Faculty of Medicine of Ribeirão Preto, University of São Paulo, Avenida Bandeirantes, 3900, Monte Alegre, 14049-900 Ribeirão Preto, SP Brazil; Department of Pathology, Faculty of Medicine of Ribeirão Preto, São Paulo University, Ribeirão Preto, SP Brazil; Department of Surgery, Faculty of Medicine of Ribeirão Preto, University of São Paulo, Ribeirão Preto, SP Brazil; Department of Physiology, Faculty of Medicine of Ribeirão Preto, São Paulo University, Ribeirão Preto, SP Brazil

**Keywords:** Urinary monocyte chemoattractant protein-1 (uMCP-1), Macrophages, Kidney transplantation, Renal failure

## Abstract

**Background:**

Inflammatory cell infiltration and residual areas of fibrosis in kidneys after renal transplantation can lead to functional abnormalities with long-term implications.

**Objectives:**

The aim of this study was to determine urinary monocyte chemoattractant protein-1 (uMCP-1) levels, relative cortical interstitial area (RCIA), and cortical tubulointerstitial macrophage infiltration in renal transplant patients with delayed graft function (DGF) and their possible correlation with graft outcome.

**Design:**

Patients were followed after biopsies for one year, and their renal function and structure were evaluated, as well as parameters of inflammatory process.

**Setting:**

Clinical Hospital of the School of Medicine of Ribeirão Preto.

**Patients:**

Twenty-two cadaveric kidney transplant recipients with DGF were followed for one year.

**Measurements:**

Renal function, RCIA, macrophages infiltration and uMCP-1 levels were evaluated.

**Methods:**

Renal function was evaluated by plasma creatinine levels. RCIA was determined by morphometry. Immunohistochemical staining of macrophages was performed using an anti-CD68 monoclonal antibody. uMCP-1 levels were determined using a human MCP-1/CCL2 immunoassay kit.

**Results:**

There was a significant increase in uMCP-1 levels in transplant patients compared with controls (*p* < 0.001). RCIA was 7.1% (6.4 to 9.2; median and 25th to 75th percentiles) in controls and 37.1% (28.1 to 43.7) in patients with kidney transplants (*p* < 0.001). The patients who presented with a higher RCIA in the first biopsy showed higher levels of plasma creatinine one year after transplantation (r = 0.44; *p* < 0.05). The number of tubulointerstitial macrophages per 0.10 mm^2^ grid field was higher in the renal cortex of transplant patients compared with the controls (19.4 (9.0 to 47.1) vs. 2.5 (1.8 to 3.4), *p* < 0.001). There was also a positive correlation between the RCIA and the number of tubulointerstitial macrophages in the renal cortex of these patients (r = 0.49; *p* < 0.001).

**Limitations:**

The number of patients studied was relatively small and may not be reflecting outcomes over a larger spectrum of kidney cadaveric transplants.

**Conclusions:**

Our results demonstrate increased levels of uMCP-1 in transplant patients with DGF, in addition to increased tubulointerstitial macrophage infiltration and RCIA, which could predict the outcome of renal function in these patients.

## Abrégé

**Contexte:**

L’infiltration de cellules inflammatoires et la présence de zone de fibrose résiduelle, après la transplantation rénale, peuvent entraîner des anomalies fonctionnelles ayant des incidences à long-terme.

**Objectifs:**

Le but de cette étude était de déterminer, chez les patients transplantés avec retard de fonctionnement du greffon (RFG), les taux urinaires de protéine chimiotactique monocytaire-1 (uMCP-1), la zone relative de l’interstitium cortical, et l’infiltration tubulo-interstitielle de macrophages dans le cortex rénal afin d’évaluer la possible corrélation de ses informations et de l’évolution de la transplantation.

**Type d’étude:**

Les patients ont été suivis pendant un an, après biopsie, avec évaluations stucturelle et fonctionnelle, et évaluation des paramètres du processus inflammatoire.

**Contexte:**

Hôpital clinique de l’école de médecine de Ribeirão Preto.

**Patients:**

Vingt-deux patients ayant reçu un greffon rénal cadavérique avec RFG ont été suivis pendant un an.

**Mesures:**

La fonction rénale, l’infiltration de macrophages et les taux d’uMCP-1 ont été évalués.

**Méthodes:**

La fonction rénale a été évaluée en utilisant les concentrations sériques de créatinine. Une coloration immunohistochimique des macrophages par anticorps monoclonal anti-CD68 a été effectuée. Les concentrations d’uMCP-1 ont été déterminées en utilisant les tests immunologiques de MCP-1/CCL2 humaine.

**Résultats:**

Les concentrations d’uMCP-1 étaient significativement plus élevées chez les patients transplantés que chez les sujets du groupe témoin (*p* < 0.001). Les médianes de la zone relative de l’interstitium cortical étaient de 7,1% (25ème au 75ème percentiles : de 6,4% à 9,2%) pour le groupe témoin et de 37,1% (de 28,1% à 43,7%) chez les patients transplantés rénaux (*p* < 0.001). Les patients ayant une la zone relative de l’interstitium cortical plus grande au moment de la première biopsie présentaient une concentration plasmatique de créatinine plus grande un an après la transplantation (r = 0.44; *p* < 0.05). Le nombre de macrophages dans l’espace tubulo-interstitiel, par champs de 0,10 mm^2^, était plus élevé dans le cortex rénal des patients transplantés que chez le groupe témoin (19,4 (de 9.0 à 47.1) et 2,5 (de 1.8 à 3,4), *p* < 0.001). Il existait également une corrélation positive entre la zone relative de l’interstitium cortical et le nombre de macrophages dans l'espace tubulo-interstitiel du cortex rénal de ces patients (r = 0.49; *p* < 0.001).

**Limites de l’étude:**

l’échantillon étudié était relativement petit et ne représente pas nécessairement les résultats d’un échantillon plus vaste constitué de patients transplantés avec greffon rénal cadavérique.

**Conclusions:**

Nos résultats démontrent des concentrations d’uMCP-1 plus élevées chez les patients avec RFG, accompagnées d’une infiltration de macrophages dans l’espace tubulo-interstitiel et d’une zone relative de l’interstitium cortical plus grandes, faits qui pourraient prédire l’évolution de la fonction rénale chez ces patients.

## What was known before

It has been observed that recovery of renal function after cadaveric transplantation with delayed graft function (DGF) is typically slow and may be incomplete. Increased monocyte chemoattractant protein-1 (MCP-1) levels have been observed in urine from patients during acute renal graft rejection compared with those with stable graft function. We found in previous studies that there was a large increase in α-smooth muscle-actin (α-SMA), fibronectin, and endothelin expression in the tubulointerstitial compartment of kidneys from patients with acute tubular necrosis after renal transplantation, which was associated with increased urinary levels of transforming growth factor β (TGF-β). These findings may explain the impaired recovery of renal function observed in these patients.

## What this adds

This study shows that increases in the urinary levels of MCP-1, relative cortical interstitial area (RCIA), and macrophage infiltration in the renal cortex could predict the evolution of renal lesions in patients with acute tubular necrosis following renal transplantation with DGF.

## Background

Renal function recovery after cadaveric transplantation with DGF is normally slow, occurring after 4–6 weeks, and may be incomplete because of acute tubular necrosis and/or rejection that lead to significant loss of nephron mass [1]. Previous studies have demonstrated persistent areas of fibrosis after renal function recovery following acute tubular necrosis [2,3]. Interstitial fibrosis is a complex process that involves the interaction between cells and cytokines and results in the accumulation of fibroblasts and extracellular matrix (ECM) in the interstitial space [4-8]. In a previous study, we demonstrated that there was a dramatic increase in the expression of α-smooth muscle-actin (α-SMA), fibronectin and endothelin in the tubulointerstitial compartment of the kidneys from patients with acute tubular necrosis post-renal transplantation. These findings were associated with higher levels of nuclear factor-κB (NF-κB) and phosphorylated c-jun N-terminal kinase (p-JNK) in tubular and interstitial cells from the renal cortex of these patients [9]. NF-κB and p-JNK participate in the progression of renal lesions in several experimental models of nephropathy by inducing the synthesis and release of inflammatory substances, promoting macrophage infiltration and cell transdifferentiation [9]. We also detected an increase in urinary transforming growth factor-β (TGF-β) excretion in transplant patients with DGF who received a cadaveric kidney. TGF-β is considered by most researchers the principal profibrogenic cytokine [6,8]. TGF-β can transform fibroblasts, tubular and mesangial cells into myofibroblasts that start to express α-SMA and have increased production of several ECM proteins [5].

Macrophages contribute to renal injury by releasing cytokines (TGF-β, endothelin, interleukin 1), angiotensin II and free radicals as well by producing several ECM components. Macrophages also play an important role in tissue homeostasis and remodeling, and are potent immune regulators [10]. Macrophages accumulate in the renal tissue during the early stages of ischemia-reperfusion injury and have a pivotal role in both innate and adaptive responses to the graft [11].

Monocyte chemoattractant protein-1 (MCP-1) is a potent proinflammatory chemokine that acts by binding to the C-C chemokine receptor 2 (CCR2) [12]. MCP-1 mediates changes in the shape, expansion and subsequent transendothelial migration of monocytes/macrophages [12]. MCP-1 has a strong chemotactic action on macrophages and has been associated with macrophage infiltration and injury in several renal diseases [13]. These data suggest that MCP-1 may be useful as a biomarker for monitoring the outcome of allografts in patients with DGF.

The aim of this study was to determine urinary MCP-1 (uMCP-1) levels, macrophage infiltration and RCIA of patients who underwent renal transplantation with DGF and their possible correlation with graft outcome.

## Methods

### Subjects

The study was approved by the Human Ethical Committee of the Clinical Hospital from School of Medicine of Ribeirão Preto (Protocol no 154405/2005). All participants provided written informed consent. Twenty-nine patients who underwent cadaveric renal transplantation and had DGF over the course of a two-year follow-up period were studied. The study took place in the Clinical Hospital of the School of Medicine of Ribeirão Preto. After biopsies were taken, the patients were followed for one year, and their renal function was evaluated by measuring plasma creatinine levels. Fresh urine was collected up to two days before or after renal biopsy to measure uMCP-1 levels. The patients with DGF were defined as those who required dialysis after transplantation.

Preserved areas of normal renal tissue from 10 patients undergoing nephrectomy for the localization of renal tumors were used as controls (n = 10 patients). This renal tissue was obtained from the macroscopically normal areas of the kidney located at some distance from the neoplastic process. After excluding patients with a history of hypertension or diabetes, controls were selected randomly after matching for age and sex, and confirming that renal tissue did not have any histological changes (interstitial fibrosis, tubular atrophy, inflammatory cell infiltration or glomerulosclerosis). The age of control patients ranged from 35 years to 55 years (mean, 48 years), and included seven males and three females. All of the control subjects were normotensive and had normal levels of plasma creatinine and 24 h proteinuria levels (<150 mg).

### Histology and morphometry

Renal biopsies were obtained from patients only upon clinical indication. If the renal function of patients did not improve, we repeated the biopsies to check if there was a change in the histological kidney pattern (Table 1). Nineteen patients underwent the first renal biopsy in the first three months following surgery and seventeen of these patients underwent more than one biopsy because of a lack of improvement of renal function (median = 2.5; maximum 4, minimum 1). Renal tissue was fixed in Bouin’s solution for 4–6 h and processed for paraffin embedding. Four-micrometer-thick histological sections were stained with Masson’s trichrome or hematoxylin and eosin and examined by light microscopy. RCIA of the renal cortex was determined by morphometry with a light camera connected to an image analyzer (Axio Vision, Carl Zeiss, Göttingen, Germany). Fifteen consecutive grid fields measuring 0.100 mm^2^ were evaluated in the renal cortex sections of each biopsy from both patients and controls. The encircled interstitial areas were manually traced on a video screen and evaluated by computerized morphometry.

**Table 1:** **Clinical data of cadaveric renal transplant recipients with delayed graft function**

	**Age (years)**	**Time on dialysis (months)**	**Time of cold ischemia (h)**	**Length of DGF (days)**	**Plasma creatinine levels after 1 year (μmol/L)**
Mean	47 ± 12.0	41.7 ± 34.8	28.2 ± 5.5	16.9 ± 13.2	144.9 ± 43.3
Range	27-71	03-132	16-38	0-46	79.5-229.8

Data are expressed as means ± SD.

### Immunohistochemistry for detection of macrophages

Kidney biopsies from 10 control individuals and 53 biopsies from the 22 patients who underwent cadaveric kidney transplantation with DGF were evaluated. The biopsy tissues were embedded in paraffin and sections stained using an indirect immunoperoxidase technique. Immunohistochemical studies were performed using a monoclonal mouse antibody to an antigen present in the cytoplasm of human monocytes and macrophages (CD68, clone KP1, IgG_1_, DAKO, Glostrup, Denmark) as the primary antibody. Briefly, serial 4-μm-thick sections were treated with 0.01 M citrate buffer (pH 6.0) in a pressure cooker for 40 min. The sections were then incubated with CD68 antibody (1:200) for 2 h at room temperature, followed by incubation with the NovoLink™ Max Polymer Detection System–Post Primary Block (Novocastra Laboratories Ltd., Newcastle Upon Tyne, UK). After three washes with Tris buffer containing Tween 20 (0.05%), the sections were incubated with the NovoLink polymer for 30 min at 37°C. Chromogen development was performed with 3,3′-diaminobenzidine (DAB; Sigma Chemical Co., St Louis, MO, USA), and the sections were counterstained with Harris’ hematoxylin, dehydrated and mounted. The negative controls were prepared by omitting the primary antibody in the reaction.

To evaluate the macrophage numbers in areas of the cortical tubulointerstitium (TI), at least 15 consecutive fields were measured. The arithmetic mean of the number of macrophages/0.10 mm^2^ of the TI area of all of the fields examined was used to express the number of macrophages in the renal cortical interstitium of each kidney. The number of macrophages/glomerular tuft (GT) area was reported as the median of the number of macrophages/GT for each biopsy.

### Urinary MCP-1 determination

Fresh urine was collected up to 2 days before or after the renal biopsy and centrifuged to remove cells and debris. Aliquots were stored at −70°C after the addition of 1 N NaOH (to reach a pH between 7.0 and 8.0) and 10 μl of 0.1 M phenylmethylsulfonyl fluoride protease inhibitor (Sigma-Aldrich Chemie GmbH, Steinheim, Germany). uMCP-1 levels were determined with the Quantikine Human MCP-1/CCL2 Immunoassay kit (R&D Systems, Minneapolis, MN, USA). The uMCP-1 values obtained were normalized to the amount of creatinine in the urine sample and reported as the ratio of uMCP-1 (pg/ml)/urinary creatinine (mg/ml). Urine samples from 10 healthy individuals were used as controls. The urinary biomarker measurements in the controls were performed in the same period used for the patients in this study.

### Statistical analysis

Data are reported as medians plus percentiles (25th to 75th). Groups were compared using the Mann–Whitney *U* Test. Correlation coefficients for regression analysis were determined using the Spearman coefficient (r) for nonparametric data. Statistical analyses were performed using Graph Pad Prism version 5.0 for Windows, Graph Pad Software, San Diego, California, USA. In all cases, a P-value of *p* < 0.05 was considered statistically significant.

## Results

### Subjects

Twenty-nine patients (18 males and 11 females) who underwent renal cadaveric transplantation and had DGF were initially enrolled in the study, although only twenty-two patients were studied. Seven patients (4 males and 3 females) were excluded from the study because of either the loss of the graft, transfer to another center or death before the first year of follow up. Twenty patients started immunosuppression with sodium mycophenolate and tacrolimus, fourteen received induction therapy with basiliximab and nine changed from tacrolimus to sirolimus during the first year post-transplantation because of suspicion of nephrotoxicity or chronic allograft nephropathies. Two patients started immunosuppression with cyclosporine A and basiliximab, and both converted to sirolimus during the first year post-transplant. The clinical data from the 22 patients studied are shown in Table 1.

### Histology

A total of 53 biopsies were examined, with a median of 2.5 biopsies (1.7 to 5.0; median and 25th to 75th percentiles) per patient. Light microscopy showed morphological features characteristic of acute tubular necrosis (defined as tubular cell necrosis and tubular lumen dilation) in 18 biopsies from 9 patients (Banff 6), features of nephrotoxicity due to drugs in 9 biopsies from 9 patients (Banff 6), chronic alterations in the graft in 17 biopsies from 13 patients (Banff 5; 12 grade I, 2 grade II and 3 grade III), normal tissue in 8 biopsies from 5 patients, and acute cellular rejection in 1 biopsy from 1 patient (Table 2). Most of the biopsies had unchanged glomerular morphology, and the mean number of glomeruli in each biopsy was 14.3 ± 9.0. The median time of biopsy after transplantation was 47 (6 to 126) d.

**Table 2:** **Histological data obtained from light microscopy of 55 patient biopsies**

**Morphological features**	**Number of biopsies**	**Number of patients**
Acute tubular necrosis	18	7
Suggestive of nephrotoxicity due to drugs	9	9
Chronic nephropathy of the graft	17	13
Acute cellular rejection	1	1
Normal tissue	8	5

### Morphometry

**Figure 1:**
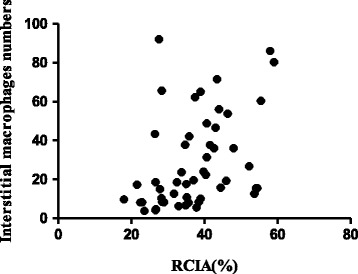
**Correlation between the relative cortical interstitial area (RCIA) and interstitial macrophages in the renal cortex tubulointerstitial area of renal cadaveric transplant recipients with delayed graft function (DGF).** (r = 0.49; *p* < 0.001).

An increase in RCIA was observed in kidney transplant patients with DGF. RCIA was 7.1% (6.4 to 9.2) in the control group and 37.1% (28.1 to 43.7) in the patients with kidney transplants (*p* < 0.001). A higher RCIA in the first biopsy was correlated with higher serum creatinine levels one year after the transplant (r = 0.44; *p* < 0.05). There was also a correlation between the RCIA and the number of interstitial macrophages (r = 0.49; *p* < 0.001) (Figure 1). We found in previous studies that an increase in RCIA is correlated with inflammation and fibrosis, which contributes to renal disease progression [9].

### Urinary MCP-1 levels

**Figure 2:**
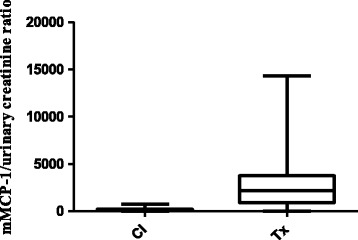
**uMCP-1 (pg/ml)/urine creatinine (mg/100 ml) ratio of the control individuals and renal cadaveric transplant recipients with delayed graft function (DGF).** Values are expressed as medians and percentiles (25th; 75th), ***p < 0.001.

There was a significant increase in the uMCP-1 levels of transplant patients with DGF compared with controls (*p* < 0.001, Figure 2). However, there was no difference in uMCP-1 levels between patients with histological findings of acute tubular necrosis (n = 9) and patients with chronic alterations (n = 13).

### Immunohistochemical studies

**Figure 3:**
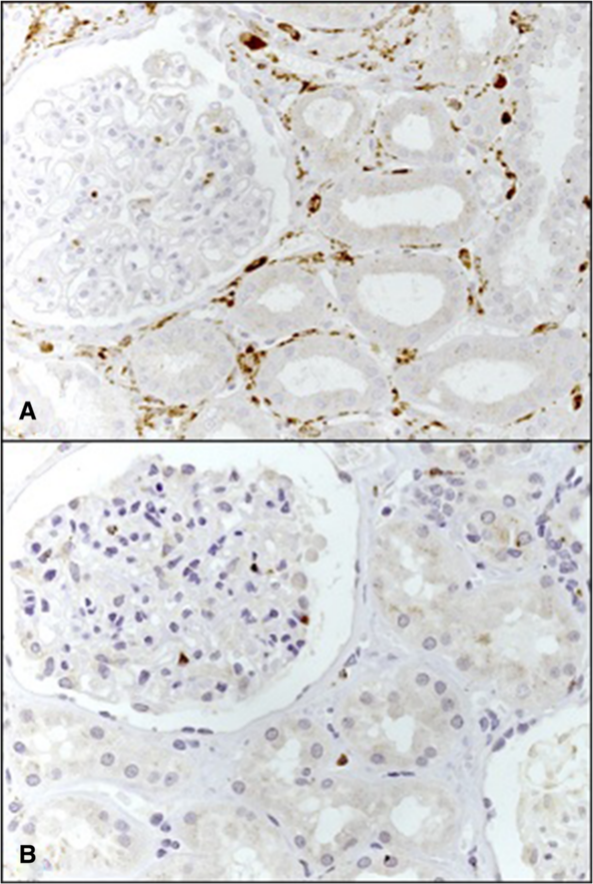
**Immunolocalization of CD68** ^**+**^ **macrophages in the renal cortex of kidneys (A) from a renal cadaveric transplant recipient with delayed graft function and (B) a control individual.**

**Figure 4:**
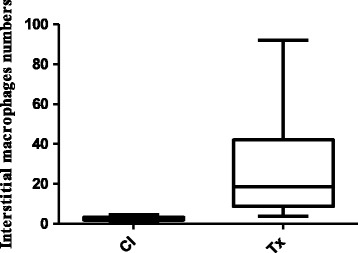
**Macrophage numbers in the tubulointerstitial area of the renal cortex from the control individuals and renal cadaveric transplant recipients with delayed graft function.** Values are expressed as medians and percentiles (25th; 75th), ***p < 0.001.

There was a significant increase in the number of tubulointerstitial macrophages in the renal cortex in transplant patients compared with controls (*p* < 0.001, Figure 3). The number of tubulointerstitial macrophages per 0.100 mm^2^ was 2.5 (1.8 to 3.4) in controls and 19.4 (9.0 to 47.1) in the cortex of kidney transplant patients (Figure 4). There was no difference in the number of macrophages in the glomerular tufts of the kidney biopsies from the transplant patients compared with the control individuals.

## Discussion

MCP-1/chemokine (C-C) motif ligand 2 (CCL2) is a potent proinflammatory chemokine that acts by binding to the C-C chemokine receptor 2 (CCR2). MCP-1 mediates changes in the transendothelial migration of monocytes/macrophages [12], has a strong chemotactic action on macrophages, and has been shown to promote macrophage infiltration and injury in several renal diseases [13]. In animal models, blocking MCP-1/CCL2 or CCR2 is associated with reduced interstitial macrophage infiltration and tubulointerstitial damage [14-16]. We found a significant increase in uMCP-1 in patients who underwent cadaveric transplantation with DGF compared with controls, suggesting the presence of active renal disease and inflammatory processes, especially in the early days post-transplantation when the majority of biopsies were obtained. However, a wide individual variability in the uMCP-1 concentration was present among the patients. The excretion of MCP-1 most likely varies according to the renal pathology and the inflammatory activity.

The increase in MCP-1 in the urine of transplant patients with DGF was associated with a significant increase in tubulointerstitial macrophages and RCIA in these patients compared with controls. We previously found a significant correlation between the RCIA and inflammation in the tubulointerstitium [9]. Therefore, this is an important finding that could also be associated with higher uMCP-1 levels. Eardley et al. [17] observed a close association between albuminuria, urinary MCP-1/CCL2 and interstitial macrophage infiltration with *in situ* damage and the clinical outcomes of patients with different glomerular diseases. Dantas et al. [18] showed increased uMCP-1 correlated with proteinuria and interstitial macrophage infiltration in patients with glomerulopathies. An increase in MCP-1 was also observed by Dubinsk et al. [19] and Prodjosudjadi et al. [20] in the urine from patients during acute renal graft rejection compared with stable graft function. The authors suggested that the increase in urinary excretion of MCP-1 is most likely the result of local production by tubular epithelial cells. MCP-1 produced locally may, at least in part, be responsible for the influx of macrophages into the interstitium during graft rejection.

The presence of macrophages in the tubulointerstitial area of the renal cortex within the transplanted kidney has been documented throughout all stages of kidney transplantation: ischemia-reperfusion injury, kidney recovery and establishment of function, the maintenance phase and chronic allograft nephropathy [11]. The roles played by macrophages include the promotion of inflammation in response to ischemia-reperfusion injury, presentation of donor antigens to primed recipient T cells, mediation of kidney damage during acute rejection, clearance of cellular debris, modulation of ECM, promotion of fibrosis and regulation of immune responses [11]. Macrophages can contribute to the innate immune response in renal ischemia-reperfusion injury, causing cell damage and inflammation. However, macrophages can also promote healing and repair as the graft recovers from acute insults and can be involved in the detrimental development of interstitial fibrosis and tubular atrophy [11]. We did not observe any differences between the numbers of macrophages in the glomerular tufts of kidney biopsies from the transplant patients compared with the control individuals. This finding shows that inflammatory process is located only in tubule interstitial area from the kidney of these patients.

Resident and infiltrating macrophages play a central role in innate immune protection, through both the clearance of infective pathogens and repair of tissue injury that occurs, in part, as a consequence of this response [21,22]. Ischemia and reperfusion results in apoptosis and necrosis [23], and the free radicals produced by damaged tissues may induce the release of inflammatory substances, such as endothelin, and chemotactic factors for both macrophages (MCP-1) and lymphocytes [24-26]. Macrophages are able to produce profibrogenic cytokines (TGF-β, PDGF, endothelin) and angiotensin, contributing to renal fibrosis and impairing renal function recovery [5,9,27]. Upon cytokine stimulation, macrophages and renal resident cells may be transformed into myofibroblasts, express α-SMA and start to produce more collagen and other ECM components, such as fibronectin [5,9,28]. Additionally, macrophages and resident cells transformed into myofibroblasts after activation became capable of synthesizing TGF-β at different stages during the development of renal fibrotic lesions [29].

Previously, we demonstrated that the NF-κB complex was activated in the renal tubular and interstitial cells of transplanted kidneys with DGF [9]. NF-κB activation may play an important role by inducing the synthesis of inflammatory substances, such as cytokines, growth factors and chemotactic factors for macrophages and monocytes that provoke renal damage in renal and extra-renal cells. Chronic allograft alterations, characterized by interstitial fibrosis, tubular atrophy and a decrease in renal function, are the major cause of graft loss [11]. The pathogenesis of these alterations remains unclear; however, inflammation is a consistent feature [30]. Experimental and clinical studies provide supportive evidence that macrophages contribute to the development of kidney disease progression [11]. Pilmore et al. [31] found that the degree of macrophage infiltration in early biopsies was predictive of the subsequent development of alterations in renal function and structure. We have shown that there was a correlation between the RCIA and renal function one year after transplantation and between the RCIA and macrophage infiltration in the renal cortex. Despite the immunosuppressive agents used by the transplant patients, macrophage infiltration and urinary MCP-1 excretion were major features within the transplanted kidney.

Delayed graft function after renal transplantation remains a problem, and patients with DGF often require dialysis in the first days post-transplant. DGF includes a spectrum of clinical manifestations ranging from borderline function to a complete absence of graft function [32]. Furthermore, cold ischemia/reperfusion is strongly associated with DGF [33].

Our study has some limitations, as we cannot exclude the influence of the renal tumor on the preserved area of nephrectomized kidneys used as the control group. The inflammation caused by the tumor cells may also be present in preserved areas of kidney biopsy. However, there was no histopathological or immunohistochemical evidence of this effect in the control biopsies. Moreover, this type of renal tissue has been used as the control tissue in multiple studies [9,34]. The best control renal tissue would be from renal transplant patients without DGF; however, we do not routinely biopsy these patients.

## Conclusion

In conclusion, the increase in uMCP-1 levels, as well as macrophage infiltration in the renal cortex interstitial area and RCIA, may help to predict the outcome of renal function in cadaveric renal transplant recipients with DGF. However, this study evaluated only a small number of patients. Future studies with larger patient cohorts are required to determine how these parameters reflect outcomes over a larger spectrum of kidney cadaveric transplants.

## Abbreviations

MCP-1: Monocyte chemoattractant protein-1
uMCP-1: Urinary monocyte chemoattractant protein-1
RCIA: Relative cortical interstitial area
DGF: Delayed graft function
TGF-β: Transforming growth factor beta
α-SMA: Alpha smooth muscle actin
ECM: Extracellular matrix
NF-κB: Nuclear factor kappa B
JNK: c-jun N-terminal kinase
CCR2: C-C chemokine receptor 2
GT: Glomerular tuft

## References

[1] Lechevallier E, Dussol B, Luccioni A, Thirion X, Vacher-Copomat H, Jaber K (1998). Posttransplantation acute tubular necrosis: risk factors and implications for graft survival. Am J Kidney Dis.

[2] Houghton DC, English J, Bennett WM (1988). Chronic tubulointerstitial nephritis and renal insufficiency associated with long-term “subtherapeutic” gentamicin. J Lab Clin Med.

[3] Pagtalunan ME, Olson JL, Tilney NL, Meyer TW (1999). Late consequences of acute ischemic injury to a solitary kidney. J Am Soc Nephrol.

[4] Desmouliere A, Geinoz A, Gabbiani F, Gabbiani G (1993). Transforming growth factor-beta 1 induces alpha-smooth muscle actin expression in granulation tissue myofibroblasts and in quiescent and growing cultured fibroblasts. J Cell Biol.

[5] Eddy AA (2000). Molecular basis of renal fibrosis. Pediatr Nephrol.

[6] el Nahas AM, Muchaneta-Kubara EC, Zhang G, Adam A, Goumenos D (1996). Phenotypic modulation of renal cells during experimental and clinical renal scarring. Kidney Int.

[7] Forbes JM, Leaker B, Hewitson TD, Becker GJ, Jones CL (1999). Macrophage and myofibroblast involvement in ischemic acute renal failure is attenuated by endothelin receptor antagonists. Kidney Int.

[8] Roelofs M, Faggian L, Pampinella F, Paulon T, Franch R, Chiavegato A (1998). Transforming growth factor beta1 involvement in the conversion of fibroblasts to smooth muscle cells in the rabbit bladder serosa. Histochem J.

[9] Moyses Neto M, Costa RS, Volpini RA, Garcia TM, Rodrigues FF, Coimbra TM (2004). Interstitial alterations in renal cortex in acute tubular necrosis (ATN) post-renal transplantation and in patients with ATN not related to renal transplant. Clin Transplant.

[10] Ricardo SD, van Goor H, Eddy AA (2008). Macrophage diversity in renal injury and repair. J Clin Invest.

[11] Chadban SJ, Wu H, Hughes J (2010). Macrophages and kidney transplantation. Semin Nephrol.

[12] Weber KS, von Hundelshausen P, Clark-Lewis I, Weber PC, Weber C (1999). Differential immobilization and hierarchical involvement of chemokines in monocyte arrest and transmigration on inflamed endothelium in shear flow. Eur J Immunol.

[13] Tang WW, Qi M, Warren JS (1996). Monocyte chemoattractant protein 1 mediates glomerular macrophage infiltration in anti-GBM Ab GN. Kidney Int.

[14] Kitagawa K, Wada T, Furuichi K, Hashimoto H, Ishiwata Y, Asano M (2004). Blockade of CCR2 ameliorates progressive fibrosis in kidney. Am J Pathol.

[15] Tang WW, Qi M, Warren JS, Van GY (1997). Chemokine expression in experimental tubulointerstitial nephritis. J Immunol.

[16] Wada T, Furuichi K, Sakai N, Iwata Y, Kitagawa K, Ishida Y (2004). Gene therapy via blockade of monocyte chemoattractant protein-1 for renal fibrosis. J Am Soc Nephrol.

[17] Eardley KS, Zehnder D, Quinkler M, Lepenies J, Bates RL, Savage CO (2006). The relationship between albuminuria, MCP-1/CCL2, and interstitial macrophages in chronic kidney disease. Kidney Int.

[18] Dantas M, Romao EA, Costa RS, dos Reis MA, Vieira Neto OM, Ribeiro RA (2007). Urinary excretion of monocyte chemoattractant protein-1: a biomarker of active tubulointerstitial damage in patients with glomerulopathies. Kidney Blood Press Res.

[19] Dubinski B, Boratynska M, Kopeé W, Szyber P, Patrzalek D, Klinger M (2008). Activated cells in urine and monocyte chemotatic peptide-1 (MCP-1) - sensitive rejection markers in renal graft recipients. Transplantl Immunol.

[20] Prodjosudjadi W, Daha MR, Gerritsma JS, Florijn KW, Barendregt JN, Bruijn JA (1996). Increased urinary excetion of monocyte chemoattractant protein-1 during acute renal allograft rjection. Nephrol Dial Transplant.

[21] Sean Eardley K, Cockwell P (2005). Macrophages and progressive tubulointerstitial disease. Kidney Int.

[22] Rodriguez-Iturbe B, Pons H, Herrera-Acosta J, Johnson RJ (2001). Role of immunocompetent cells in nonimmune renal diseases. Kidney Int.

[23] Thadhani R, Pascual M, Bonventre JV (1996). Acute renal failure. N Engl J Med.

[24] Schlondorff D (1995). The role of chemokines in the initiation and progression of renal disease. Kidney Int.

[25] Summan M, Warren GL, Mercer RR, Chapman R, Hulderman T, Van Rooijen N (2006). Macrophages and skeletal muscle regeneration: a clodronate-containing liposome depletion study. Am J Physiol Regul Integr Comp Physiol.

[26] Vandivier RW, Henson PM, Douglas IS (2006). Burying the dead: the impact of failed apoptotic cell removal (efferocytosis) on chronic inflammatory lung disease. Chest.

[27] Kliem V, Johnson RJ, Alpers CE, Yoshimura A, Couser WG, Koch KM (1996). Mechanisms involved in the pathogenesis of tubulointerstitial fibrosis in 5/6-nephrectomized rats. Kidney Int.

[28] Groma V (1998). Demonstration of collagen type VI and alpha-smooth muscle actin in renal fibrotic injury in man. Nephrol Dial Transplant.

[29] Eddy AA (2005). Progression in chronic kidney disease. Adv Chronic Kidney Dis.

[30] Nankivell BJ, Borrows RJ, Fung CL, O’Connell PJ, Allen RD, Chapman JR (2003). The natural history of chronic allograft nephropathy. N Engl J Med.

[31] Pilmore HL, Painter DM, Bishop GA, McCaughan GW, Eris JM (2000). Early up-regulation of macrophages and myofibroblasts: a new marker for development of chronic renal allograft rejection. Transplantation.

[32] McLaren AJ, Jassem W, Gray DW, Fuggle SV, Welsh KI, Morris PJ (1999). Delayed graft function: risk factors and the relative effects of early function and acute rejection on long-term survival in cadaveric renal transplantation. Clin Transplant.

[33] Ojo AO, Wolfe RA, Held PJ, Port FK, Schmouder RL (1997). Delayed graft function: risk factors and implications for renal allograft survival. Transplantation.

[34] Segerer S, Hughes E, Hudkins KL, Mack M, Goodpaster T, Alpers CE (2002). Expression of the fractalkine receptor (CX3CR1) in human kidney diseases. Kidney Int.

